# Successful 3D imaging of cleared biological samples with light sheet fluorescence microscopy

**DOI:** 10.1083/jcb.202307143

**Published:** 2023-10-17

**Authors:** Elise Delage, Thomas Guilbert, Frank Yates

**Affiliations:** 1 CellTechs Laboratory, SupBiotech, Villejuif, France; 2 Service d’Etude des Prions et des Infections Atypiques, Institut François Jacob, Commissariat à l’Energie Atomique et aux Energies Alternatives, Université Paris Saclay, Fontenay-aux-Roses, France; 3 Institut Cochin, Institut national de la santé et de la recherche médicale (U1016), Centre National de la Recherche Scientifique (UMR 8104), Université de Paris (UMR-S1016), Paris, France

## Abstract

In parallel with the development of tissue-clearing methods, over the last decade, light sheet fluorescence microscopy has contributed to major advances in various fields, such as cell and developmental biology and neuroscience. While biologists are increasingly integrating three-dimensional imaging into their research projects, their experience with the technique is not always up to their expectations. In response to a survey of specific challenges associated with sample clearing and labeling, image acquisition, and data analysis, we have critically assessed the recent literature to characterize the difficulties inherent to light sheet fluorescence microscopy applied to cleared biological samples and to propose solutions to overcome them. This review aims to provide biologists interested in light sheet fluorescence microscopy with a primer for the development of their imaging pipeline, from sample preparation to image analysis. Importantly, we believe that issues could be avoided with better anticipation of image analysis requirements, which should be kept in mind while optimizing sample preparation and acquisition parameters.

## Teaser

Delage et al. provide a primer on light sheet fluorescence microscopy to guide biologists through the specific challenges of this 3D imaging technique.

## Introduction

Light sheet fluorescence microscopy (LSFM), also referred to as selective plane illumination microscopy (SPIM), has become the technique of choice for three-dimensional (3D) imaging of large biological specimens in a non-destructive approach. The fundamental principle of LSFM consists of illuminating a single plane of a fluorescent sample with a thin sheet of light. This sheet is coplanar with the focal plane of a detection lens located perpendicularly, which is used to collect the emitted fluorescence to form an enlarged image. Volumetric imaging is achieved by moving the sample through the light sheet (Olarte et al., 2018). The optical sectioning provided by decoupling illumination and detection permits a much faster acquisition compared with confocal microscopes, thus reducing photobleaching while avoiding out-of-focus signals. LSFM was popularized in the last decade with the release of commercial systems making the technology accessible to more research groups and with the improvement of tissue-clearing methods for a wide range of samples, from organoids to whole-mount organs and organisms (Dyer et al., 2022; Ueda et al., 2020a, 2020b; Vieites-Prado and Renier, 2021; Wan et al., 2019).

A typical visualization of LSFM data sets is a rotating 3D animation in which it is possible to navigate to obtain a close-up view of the structures of interest deep inside the sample at the cellular scale. Such appealing outcomes have pushed many biologists to try the technique. However, the enthusiasm aroused by the acquisition of the first 3D images often gives way to frustration at the difficulty of obtaining meaningful biological information (Watkins and St. Croix, 2018; Weiss et al., 2021). Ultimately, due to the challenges of LSFM, some researchers return to more conventional microscopy techniques, such as laser scanning confocal microscopy, spinning disk confocal microscopy, or two-photon microscopy. While 3D imaging is an undeniable advantage for many projects, it is not adapted to all biological inquiries. Implementing an LSFM imaging pipeline from sample preparation to data analysis is a tedious process, with optimizations required at every step, therefore anticipating difficulties is essential for success.

We surveyed LSFM users from various institutes and companies by means of an online questionnaire that we submitted to (1) the users of our core facility at the Commissariat à l’Energie Atomique et aux Energies Alternatives, (2) the diffusion list of the Réseau Technologique de Microscopie photonique de Fluorescence Multidimensionnelle (Centre National de la Recherche Scientifique; https://rtmfm.cnrs.fr/), which includes researchers, technicians, PhD students, and core facilities from French academic research (Centre National de la Recherche Scientifique, Institut national de la santé et de la recherche médicale, universities…). We received responses from 9 users describing themselves as “experts” of the technique, 19 as “autonomous users,” and 7 as “novices.” The participants provided information regarding the samples they analyzed (which were very diverse, including organoids, a broad variety of mouse tissues and organs, embryos, and entire organisms such as zebrafish), the tissue-clearing method they applied, the LSFM system they worked on, and the image analysis software they used. Of the 35 responses obtained, one-third declared that their experience with LSFM had not met their expectations (including four out of the nine self-declared expert users). The most frequently mentioned hindrance was the limitations of the microscope (resolution, magnification, size of the field of view (FOV), etc.). Next was the difficulty of obtaining samples that were transparent enough to image their entire volume. Finally, preserving the fluorescence of endogenous proteins such as green fluorescent protein (GFP) and analyzing the obtained data sets were cited as obstacles. Although the size of the interrogated panel is limited, the reported hurdles are consistent with the literature, including the results from a similar survey performed by another group in 2021 (Molbay et al., 2021). We are thus confident that it accurately depicts the difficulties encountered by many LSFM users.

**Figure 1.**
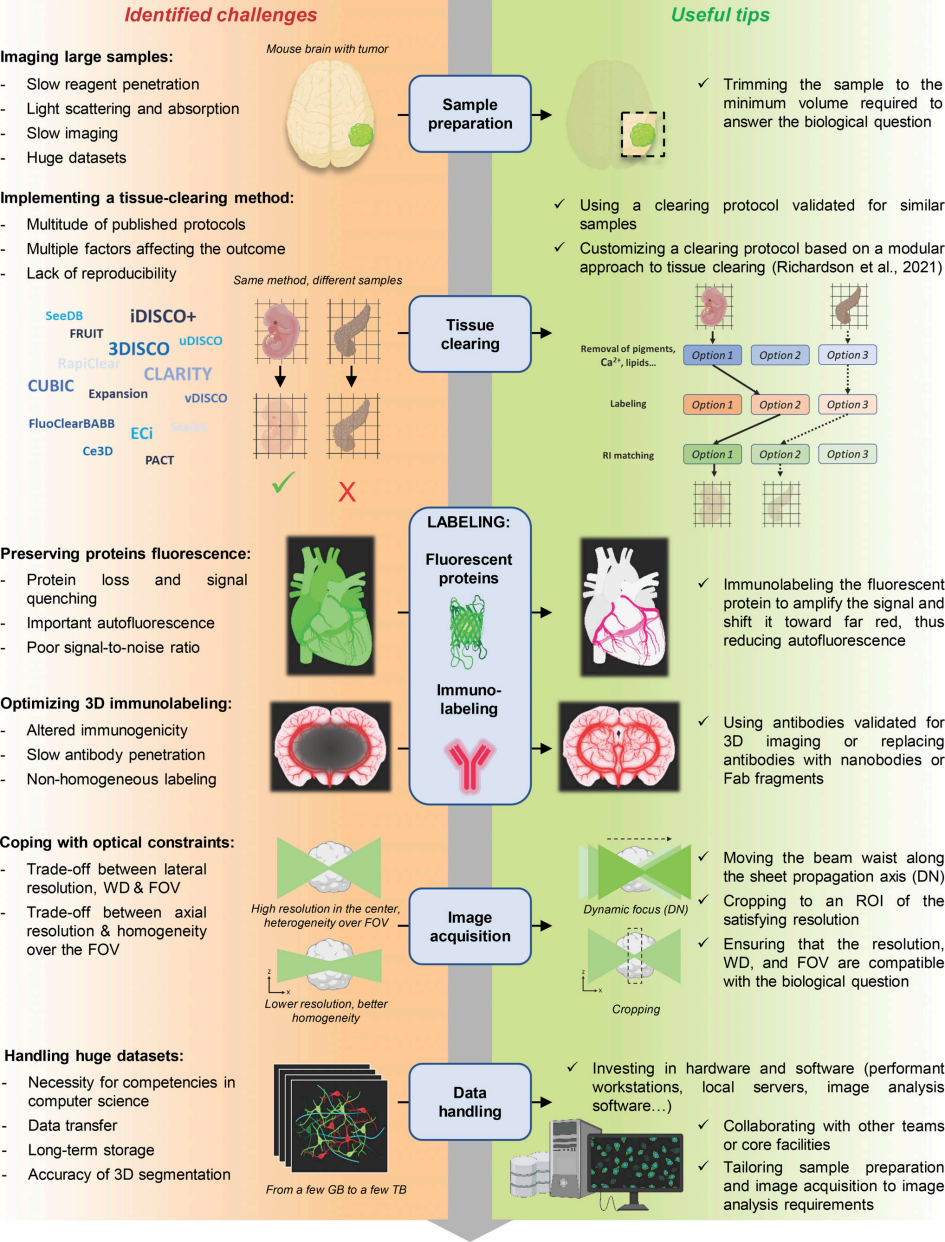
**Identified challenges and useful tips to optimize a 3D imaging pipeline from sample preparation to image acquisition.** WD: working distance; GB: gigabyte; TB: terabyte, DN: dynamic focus; ROI: region of interest. Created with Biorender.com.

We wrote this review to provide biologists tempted to embark on the adventure of LSFM imaging of cleared samples with a “survival guide” compiling the known pitfalls and useful tips gathered both from our experience as an LSFM facility and from the literature (Fig. 1). We hope this will save them valuable time and help obtain meaningful data from their acquisitions.

## Sample trimming

LSFM coupled with tissue clearing is often advertised as the best solution to image large biological samples (“large” meaning several millimeters or up to a few centimeters, depending on what the system can accommodate). Protocols for whole-mount imaging of nearly every organ in small vertebrates are easily found in the literature (Alessio and Zhang, 2021; Epp et al., 2015; Frenkel et al., 2023; Kolesová et al., 2021; Maldonado et al., 2020; Nehrhoff et al., 2016; Wu et al., 2021). Strikingly, several research teams even reported successful clearing and imaging of entire rodent bodies at single-cell resolution (Cai et al., 2019; Kubota et al., 2017; Mai et al., 2023; Pan et al., 2016; Tainaka et al., 2014). However, with larger sample sizes there are more problems at each step of the imaging pipeline. Fortunately, visualization of entire organs or organisms is not always necessary. In that case, it is highly recommended to start by trimming the fixed sample to the minimum volume before processing to tissue clearing (as any cut performed after this step is more likely to introduce asperities or physical breaks that will disturb the light sheet propagation, thus creating artifacts) (Ariel, 2018; Richardson et al., 2021). Trimming accelerates reagent penetration considerably during sample preparation and increases the probability of obtaining homogeneous labeling and clearing. In addition, no existing clearing method has been shown to fully eliminate absorption and scattering as light propagates deep into the tissues (Hobson et al., 2022; Kim et al., 2018; Wan et al., 2018; Woo et al., 2022). Smaller samples are therefore easier to image. Finally, imaging smaller samples is faster and results in smaller data sets, which is a significant advantage given the important volume of images produced by LSFM (Reynaud et al., 2015).

## Tissue clearing

The opacity of biological samples is due to light scattering caused by the high heterogeneity of refractive indices (RI) among the materials that compose biological matter. Clearing methods essentially aim to reduce this heterogeneity to allow deeper light penetration (Richardson et al., 2021; Richardson and Lichtman, 2015). Over the past two decades, a remarkable diversity of clearing protocols has been published and a few commercial kits have appeared on the market (Molbay et al., 2021; Vieites-Prado and Renier, 2021). Such an abundance of techniques can be somewhat confusing for biologists aiming to implement tissue clearing in their laboratories. In addition, reproducing these methods is rarely straightforward. Many factors can influence the outcome, including the nature of the sample and user errors due to an insufficiently detailed protocol in the material and methods section, or incubation times minimized in publication to enhance the apparent rapidity of the technique (Kim et al., 2018; Molbay et al., 2021; Weiss et al., 2021). The main lesson that can be drawn from the proliferation of clearing protocols is that one method may be better than the others in a specific context, but there is no well-defined pipeline that can be confidently applied to all types of specimens and labeling without troubleshooting. Using a method validated for similar samples (if possible, by multiple groups) will save considerable time and energy. In addition to published articles, online resources from the tissue-clearing community, such as protocols annotated with useful tips or feedback from users can be very useful (Table 1). Some imaging facilities are also starting to offer sample labeling and clearing as a service, which is particularly convenient for methods requiring specialized equipment, such as clear lipid-exchanged acrylamide-hybridized rigid imaging/immunostaining/in situ-hybridization-compatible tissue hydrogel (CLARITY).

**Table 1.** Useful resources for each step of the 3D imaging pipeline

	Resource	Description
Tissue clearing and labeling	https://idisco.info/	From the inventors of iDISCO; updated protocols and useful tips, including a list of validated antibodies.
	https://transparent-human-embryo.com/	From the inventors of 3DISCO; collection of 3D datasets of human embryos and fetuses, protocols with useful tips, including a list of validated antibodies.
	https://clearlightbiotechnologies.com/	Company founded by the inventor of CLARITY, providing consulting services for CLARITY tissue clearing, including a list of antibodies optimized for thick tissue penetration.
	http://discotechnologies.org/	Compilation of resources for protocols of the DISCO family, including didactic demonstration videos.
	http://cubic.riken.jp/	From the inventors of CUBIC; updated protocols with useful tips.
	Richardson and Lichtman (2015)	An essential reference on the physicochemical principle of the various tissue-clearing methods.
	Parra-Damas and Saura (2020)	Review article on tissue-clearing applications for the study of neuropathological processes. It includes a useful table summarizing the key features of the main tissue-clearing methods.
	Ueda et al. (2020b)	Review article on tissue-clearing applications in neuroscience. It includes a useful table compiling tissue-clearing reagents and their role in the process.
	Richardson et al. (2021)	An essential primer describing a modular approach to tissue clearing to rationally design a customized clearing protocol according to the specificities of the sample.
	Vieites-Prado and Renier (2021)	Extensive review article on tissue-clearing applications in developmental biology. It includes a useful table of tissue-clearing methods with their pros and cons, guidelines to choose an adequate method, a figure illustrating a modular vision of tissue clearing with the various reagents that can be used for each module, and a table summarizing model species analyzed with tissue clearing.
	Weiss et al. (2021)	Tutorial describing the challenges associated with tissue clearing and providing valuable recommendations to optimize sample preparation and imaging. It includes a very interesting figure illustrating classical artifacts encountered when imaging cleared samples with LSFM.
Light sheet microscopy	Ariel (2018)	This guide written for users of the LaVision Biotec Ultramicroscope II system (now Miltenyi Biotec) compiles a lot of valuable considerations and recommendations, many of which apply generally to LSFM.
	Olarte et al. (2018)	(Very) comprehensive review for readers interested in LSFM from an optics perspective. It presents guidelines for designing a customized LSFM, various system architectures, and strategies to push forward the resolution limits. It also provides a table comparing the key features of commercial LSFM systems.
	Ueda et al. (2020b)	(Already mentioned for tissue clearing.) It includes a very useful figure comparing the resolution, speed, and working distance of custom and commercial LSFM systems.
Image analysis	Reynaud et al. (2015)	A commentary on the challenges associated with LSFM and the interdisciplinarity required by the technique. It includes valuable recommendations for image processing and for handling the huge volume of data generated.
	Gibbs et al. (2021)	An essential review of LSFM image analysis software. It presents basic computing concepts required to develop an image analysis pipeline and provides guidelines to select an adequate analysis tool. It includes several useful figures, such as an illustration of the range of light-sheet data sizes, computing environments, and processing tasks and an overview of the hardware/software solutions employed by several research groups.

Clearing methods have been historically divided into two families depending on the hydrophobic or hydrophilic nature of the clearing solutions. “Hydrophobic methods” are based on sample dehydration followed by clearing in organic solvents with high RI. These methods are fast and provide high transparency, but not all of them are compatible with the observation of fluorescent proteins (Molbay et al., 2021; Vieites-Prado and Renier, 2021). “Hydrophilic methods” are based either on simple immersion in high RI solutions, hyperhydration, or hydrogel embedding to stabilize the sample structure and allow efficient delipidation using strong ionic detergents that can be combined with active transport via electrophoresis (Richardson and Lichtman, 2015; Vieites-Prado and Renier, 2021). Hydrophilic methods allow better preservation of endogenous fluorescent proteins. However, the passive approaches are time-consuming and may fail to clear larger samples, while the active ones are faster and more efficient but require specialized—and expensive—equipment. For each method, a few leading techniques have emerged and can constitute a good starting point for beginners in the field. Among hydrophobic methods, 3D imaging of solvent-cleared organs (3DISCO; Belle et al., 2014) and immunolabeling-enabled DISCO (iDISCO [Renier et al., 2014]/iDISCO+ [Renier et al., 2016]) have proved their worth in many contexts, but do not preserve protein fluorescence. Ethyl cinnamate (Klingberg et al., 2017) provides a fast, low-cost, non-toxic, and GFP-compatible alternative, at least for preliminary tests, although it may fail to achieve fully satisfying clearing of larger or lipid-rich samples. Among hydrogel-based methods, CLARITY (Chung et al., 2013) stands out for its efficacy and compatibility with multiple immunofluorescence staining, although it requires expensive equipment. Clear, unobstructed brain/body imaging cocktails and computational analysis (CUBIC; Susaki et al., 2014), based on sample hyperhydration, constitute a cheaper alternative but may imply more optimization. Excellent extensive reviews will provide the reader with detailed explanations of the various approaches and include very useful tables comparing their pros and cons (Table 1; Avilov, 2021; Parra-Damas and Saura, 2020; Richardson et al., 2021; Ueda et al., 2020a; Vieites-Prado and Renier, 2021; Woo et al., 2022). Nevertheless, while these comparisons are useful for making an initial choice, they cannot encompass the diversity of factors that will influence the quality of the outcome (Avilov, 2021; Kim et al., 2018; Weiss et al., 2021).

The initial dichotomy between hydrophilic and hydrophobic methods is becoming outdated, as some experts advocate a modular vision of the tissue-clearing process, in which modules involving organic solvents can be combined with modules involving aqueous solutions (Richardson et al., 2021; Vieites-Prado and Renier, 2021). Indeed, most protocols share common steps, which consist of fixing the sample; removing light scattering and absorbing materials such as lipids, calcium, and pigments; labeling; and optical clearing. Each of these steps could be independently optimized for a given sample and given labeling based on an informed understanding of the action of each reagent. An excellent tutorial provides an invaluable guide for biologists willing to design their customized clearing protocol (Richardson et al., 2021). While this modular approach is highly advisable when tissue clearing is central to a research project, it may discourage research teams who just want to do a quick preliminary test or have a very transient need. In this case, it is preferable to adopt the “classical” techniques mentioned above.

## Labeling

GFP and other genetically encoded proteins are invaluable tools for fluorescence imaging and, in the early 2000s, motivated the rising interest in LSFM. Various tissue-clearing protocols claim to be compatible with GFP observation (Hama et al., 2011; Hou et al., 2015; Ke et al., 2016; Pan et al., 2016; Qi et al., 2019; Schwarz et al., 2015; Susaki et al., 2014; Tainaka et al., 2018; Vieites-Prado and Renier, 2021). Nevertheless, it must be stressed that published proofs-of-concept for the retention of protein fluorescence are often based on GFP or yellow fluorescent protein expressed under a strong promoter in a sparse cell population, such as the Thy1 promoter in the brain (Ariel, 2017). Researchers applying these protocols to their own samples, with a lower and more diffuse signal, often realize to their consternation that the reality is far from the theory because partial protein loss and signal quenching cannot be avoided (Weiss et al., 2021). Furthermore, large biological samples are associated with substantial light scattering, absorption, and autofluorescence, all of which constitute prominent issues for the detection of GFP signal, typically around 510 nm, while they decrease for higher wavelengths (Ariel, 2017, 2018; Weiss et al., 2021). Obtaining a satisfying signal-to-noise ratio for GFP is therefore challenging. In addition, a compromise must often be made between fluorescence preservation and transparency, as methods gentler to fluorescent proteins are usually less efficient in tissue clearing (Vieites-Prado and Renier, 2021).

Immunolabeling offers a good alternative to obtain a strong fluorescent signal, although it is more time-consuming, as it necessitates long incubation times (usually a few days, up to a few weeks for bigger samples), and more expensive as it requires large quantities of antibodies. However, obtaining specific and homogeneous staining for large biological samples is not straightforward, even with antibodies that are routinely used for 2D immunolabeling. Antigenicity can be altered upon fixation and clearing, and another major problem resides in the very slow penetration of antibodies into tissues (Vieites-Prado and Renier, 2021; Weiss et al., 2021). A high antigen density can result in antibodies being absorbed by peripheral epitopes faster than they can diffuse through the tissue, resulting in a fluorescent “halo” at the periphery of a mostly unlabeled sample (Richardson et al., 2021; Weiss et al., 2021). Interestingly, Zwang et al. (2023) recently published an approach inspired by quantitative structure–activity relationship modeling for drug discovery to facilitate the optimization of immunolabeling protocols for thick tissues. Their strategy is based on a library of mouse brain tissues prepared by varying only one or two parameters from a baseline immunolabeling protocol that was used to determine the optimal staining conditions for a panel of antibodies. Their study highlights that, rather than the antibodies themselves, minor changes in key processing steps such as fixation, pretreatment, or clearing, deeply impact the staining quality. Moreover, they evidence that not all antibody–antigen pairs are influenced in the same way by these variations, confirming the requirement to optimize the procedure for each target. Lists of antibodies validated for specific tissue-clearing methods are published online by various research groups with strong expertise in 3D imaging (Table 1), which greatly benefit the community. Some companies also started to provide antibodies optimized for 3D immunolabeling, although their catalogs are still relatively limited to date. Protocols aiming to facilitate antibody penetration by optimizing tissue permeabilization (small-micelle-mediated human organ efficient clearing and labeling; Mai et al., 2022), controlling antibody activation (system-wide control of interaction time and kinetics of chemicals, SWITCH; Murray et al., 2015), or applying physical forces (active clarity technique-pressure related efficient and stable transfer of macromolecules into organs; Lee et al., 2016) could represent a strong improvement for some tissues, such as human biopsies. While previous approaches for imaging the entire mouse body relied on transgenic expression of fluorescent proteins, a whole-body immunolabeling protocol using standard IgG antibodies was recently developed (Mai et al., 2023). This protocol named wildDISCO (immunolabeling of wildtype mice and DISCO clearing) combines cholesterol extraction for enhanced antibody penetration with DISCO-based tissue clearing and was used to generate high-resolution whole mouse atlases available online (https://discotechnologies.org/wildDISCO/atlas/index.php). Finally, another promising approach consists of replacing classical antibodies with nanobodies or Fab fragments, which diffuse much faster through tissues, thanks to their smaller size (around 15 kD, compared with 150 kD for classical antibodies; Ariel, 2017; Cai et al., 2019; Richardson et al., 2021). However, these tools are only available for a small range of targets, and their selection and validation against new epitopes is a laborious process.

Alternatively, GFP expression associated with signal amplification based on immunolabeling is an alternative and valuable means of obtaining a strong signal while shifting the fluorescence toward the far-red channel, thus reducing autofluorescence issues (Ariel, 2017). GFP antibodies validated for 3D imaging can easily be found in the literature (Adolfs et al., 2021; Lee et al., 2016; Renier et al., 2014; Takahashi et al., 2022). Along the same line of thought, variable domain of heavy chain antibodies (nanobodies) DISCO technology (vDISCO) provides strong amplification of the fluorescent-protein signal thanks to nanobodies conjugated with bright fluorophores directed against these proteins (Cai et al., 2019).

## Image acquisition

Like tissue clearing, the literature on light sheet microscopy is prolific and somewhat confusing given the diversity of terminology and abbreviations used to designate the multiple systems and variants of the technique (Lemon and McDole, 2020). Notably, many research groups have implemented their home-built systems, with a variety of features, such as multiview imaging, dipping lenses, or closed imaging chambers (Delgado-Rodriguez et al., 2022; Olarte et al., 2018; Ueda et al., 2020b). Open-source designs are accessible online, such as OpenSPIM (Pitrone et al., 2013) or mesoSPIM (Voigt et al., 2019), offering a cheaper “do-it-yourself” alternative compared with commercial light sheets, which have appeared on the market relatively recently. While this approach is ideal for matching the specific needs of a given research project, it requires time and technical expertise, and the resulting equipment may lack versatility in its applications (Delgado-Rodriguez et al., 2022; Girstmair et al., 2016). Therefore, core facilities or other multiuser environments often prefer turnkey commercial systems, which are more user-friendly and can accommodate a wide range of biological samples and imaging media, including organic solvents. It is not in the scope of this review to detail the existing systems, but we refer the reader to excellent reviews on the subject, including interesting comparisons of the pros and cons of the various options (Table 1; Olarte et al., 2018; Ueda et al., 2020b).

Biologists unfamiliar with LSFM, who are not ready to acquire or construct a light sheet microscope, might explore collaborations with other research groups or imaging platforms that have such equipment. Since the technology is not yet widespread, they may be limited in the choice of systems available in their immediate vicinity. However, the characteristics of the available LSFM should be thoroughly considered from the very beginning of a 3D imaging project to ensure that it is suited to the desired application. For instance, while some systems are equipped with cuvettes, sample holders, and even dipping lenses designed to resist organic solvents that are used for tissue-clearing (benzyl ether, ethyl cinnamate, etc.), others are only compatible with aqueous media (Ariel, 2018; Flood et al., 2013).

**Figure 2.**
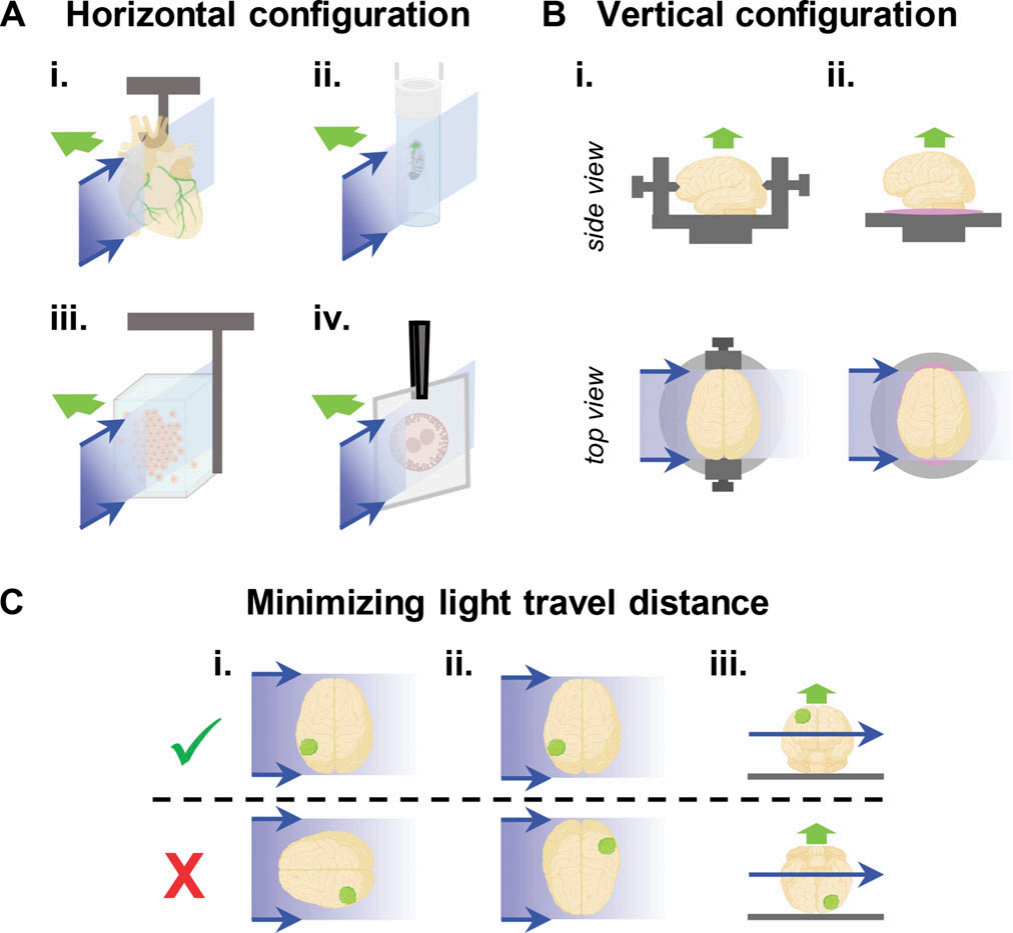
**Sample mounting in LSFM.** Blue arrows represent the light sheet propagation and green arrows the detection beam path. **(A)** Examples of sample mounting for LSFM systems with a horizontal configuration of the illumination and detection beam paths. Samples can be suspended with a hook (i), embedded in a gel cylinder (ii), immersed in a transparent container (iii), or flat mounted on a coverslip (iv). Various systems provide the option to rotate the sample along a vertical axis for multiview imaging. **(B)** Examples of sample mounting in LSFM systems with a vertical configuration of the illumination and detection beam paths. Samples can be screwed (i) or glued (ii) on a sample holder. **(C)** In systems with a vertical setup, it is crucial to minimize the light travel distance. The narrower section of the sample should be oriented in the illumination axis (i). If possible, structures of interest (here, a green tumor) should be positioned on the same side as the illumination (ii) and on the upper part of the sample (iii). Created with Biorender.com.

The physical decoupling of the illumination and detection objectives and their perpendicular arrangement, which is typical of LSFM compared with epifluorescence microscopy, entail some specific constraints concerning sample mounting. Depending on the system, the illumination and detection beam paths can either be arranged in a horizontal (e.g., ZEISS Lightsheet Z1 or the more recent Z7) or vertical setup (e.g., Miltenyi Ultramicroscope I and II or the more recent Ultramicroscope Blaze) with respect to the detection axis. For horizontal configurations, which are the most common, samples are usually hung from above in front of the objectives (Fig. 2 A). Larger samples can be suspended with a hook, a clip, or tweezers, although precautions should be taken to avoid damaging the tissues or interfering with image acquisition (Flood et al., 2013; Reynaud et al., 2008). Another classical approach consists of embedding the sample in a gel cylinder (typically, 1% low melting agarose) extruded from a tubular object such as a syringe or glass capillary. Samples that cannot be easily embedded can be placed in a suitable transparent container that can be made from gelling agents or specific polymers. In some systems, very small samples can be flat mounted on a coverslip which is held vertically. Alternatively, the sample can be supported from below for greater stability (Reynaud et al., 2008). Interestingly, different systems with a horizontal setup often provide the possibility to rotate the sample, thus allowing for multiview imaging. This is especially advantageous when imaging imperfectly cleared samples and for avoiding shadowing effects due to highly scattering objects (Hobson et al., 2022). In systems with a vertical setup, the specimen is usually stabilized with screws or glued on a sample holder (Fig. 2 B; Ariel, 2018). As for horizontal systems, live, soft, or very small samples are more conveniently mounted and positioned when embedded in a hydrogel. LSFM systems with a vertical configuration do not usually allow for sample rotation and multiview imaging. However, some of them are equipped with dual-side illumination to compensate for light sheet degradation due to light scattering as it propagates through the sample. When using one-side illumination, it is crucial to minimize the light travel distance and thus position the sample so that it is narrower in the propagation axis (Fig. 2 C; Ariel, 2018). When imaging a specific area of interest within a larger sample, its position relative to both the illumination and detection objectives must be optimized. If it is compatible with the desired view, structures of interest must be positioned on the same side as the illumination and on the upper part of the sample, which is closer to the detection lens, thus maximizing the collection of emitted photons (Fig. 2 C). It is important to keep in mind that each LSFM system comes with its specific mounting requirements and therefore may not be compatible with some applications. For instance, while plant root growth can be imaged live on systems with a horizontal arrangement, in which plantlets are grown vertically in an agarose cylinder, it cannot be imaged on systems with a vertical arrangement (Flood et al., 2013).

Most users will not need a detailed understanding of the physics and subtleties of the LSFM system that they use, but they must know what volume they can image and at what resolution. Compared to epifluorescence microscopes, for which the major constraints are associated with the main objective used for both illumination and detection, a specificity of LSFM is the further set of constraints added by the illumination lens. Most common light sheet designs are based on a cylindrical lens generating the light sheet as a Gaussian beam. The thinner the light sheet, the better the axial resolution (Dyer et al., 2022). However, this illumination beam does not have a constant thickness along its propagation axis. Rather than a sheet, it has a bowtie shape, with the narrowest point determined by the lens’s numerical aperture (NA; Fig. 1; Ariel, 2018; Hobson et al., 2022; Parthasarathy, 2018). A high NAprovides a very narrow waist in the beam, but the thickness of the optical section dramatically increases with the distance from the focal point. The resulting image may thus be resolved in the center of the FOV but blurry on the edges. Reducing the lens NA to generate a more homogeneous light sheet over the FOV may considerably increase the quality of acquisition, although it reduces the axial resolution (Ariel, 2018; Richardson et al., 2021). To avoid compromising on the NA, some systems allow moving the beam waist along the sheet propagation axis to reconstruct a final image with optimal resolution (Ariel, 2018; Weiss et al., 2021). Alternatively, the image can be cropped to a smaller region of satisfying resolution. This latter solution has several advantages. Indeed, it is also a way to get around spherical aberrations (although it can be partially corrected at the level of the optical system, the outer part of the FOV appears blurry and distorted compared with the center when using spherical lenses), and to have a homogeneous illumination along the y axis (the illumination power in this dimension follows a Gaussian curve and therefore tends to be dimmer on the edges; Ariel, 2018; Newmaster et al., 2022; Richardson et al., 2021). Tiling can then be used to acquire a larger area of the sample (Ariel, 2018; Hobson et al., 2022).

New light sheet users are often tempted to maximize the magnification and resolution of their acquisitions. However, this is not necessary nor advisable during the optimization process, when low-quality images are sufficient to evaluate the transparency and labeling of the sample. Even for optimized samples, it is preferable to reduce the magnification and resolution to the minimum necessary to answer the biological question. Acquisitions at higher magnifications with a high NA lens will be more impacted by RI mismatches within the sample (Ariel, 2018; Weiss et al., 2021). In addition, isotropic resolution across all three dimensions is critical to obtain accurate 3D reconstructions. Since the axial resolution is usually the limiting factor, this may involve reducing the resolution in x and y to the maximum resolution that can be achieved in z. Finally, imaging a large sample at high magnification and resolution inevitably increases the acquisition time and the volume of data generated, rendering the obtained images more difficult to handle (Reynaud et al., 2015).

To give an order of magnitude despite great heterogeneity, most LSFM systems offer magnification that does not exceed 20× and resolution in the micrometer range in x, y, and z (Vieites-Prado and Renier, 2021; Weiss et al., 2021). Researchers aiming to image moderately large samples at subcellular resolution should consider using a confocal microscope, which features higher magnification lenses with long working distances. Nowadays, efforts are concentrating on pushing forward the limit of resolution, by generating thinner and more homogeneous light sheets and by combining LSFM with various super-resolution techniques such as stochastic optical reconstruction microscopy, stimulated emission depletion microscopy, and structured illumination microscopy (Olarte et al., 2018). An elegant way of improving acquisition speed, spatial resolution, homogeneity, excitation strength, or compensating optical aberrations is to use adaptive techniques. Whether these techniques are physical, based on mirrors or spatial light modulators (adaptive optics; Ji, 2017; Liu et al., 2023; Olarte et al., 2018), part of a complete framework (Royer et al., 2018) or, more recently, deep learning based (Rai et al., 2023), they can significantly improve the quality of acquisitions. Excellent reviews summarizing the strategies allowing for improvement of LSFM resolution can be found in the literature (Delgado-Rodriguez et al., 2022; Fiolka, 2021; Lemon and McDole, 2020; Olarte et al., 2018; Parthasarathy, 2018; Watkins and St. Croix, 2018). However, it should be noted that most of these advanced systems are not widespread and will not be accessible to many users.

## Data handling

Once sample preparation and imaging have been optimized, the final step is to process and analyze the resulting images to get biological insight. However, this step is often insufficiently anticipated and the associated difficulties are underestimated. One of the main obstacles is the volume of the resulting data sets, which ranges from huge to colossal depending on the sample size and the acquisition parameters. To give an idea, even the simplest 3D acquisitions classically produce a few gigabytes of data. Multichannel acquisitions using tiling to image centimeter-large samples with a cellular resolution (which can be a routine demand on an LSFM platform) can produce up to a terabyte of data or more (Reynaud et al., 2015; Vieites-Prado and Renier, 2021; Weiss et al., 2021). With such huge data sets, every step becomes a challenge: data transfer from the acquisition computer to the analysis workstation, long-term data storage, as well as data visualization and analysis. Indeed, these volumes of data exceed the random access memory of most computers (Weiss et al., 2021). Biologists used to analyzing images acquired with classical microscopes on their laptops will need a high-end workstation to simply visualize their LSFM images. For the largest data sets, local servers or high-performance computing clusters are required (Gibbs et al., 2021; Reynaud et al., 2015). Without a consequent investment in both dedicated hardware and software, the added value of LSFM remains very limited. Unfortunately, while institutions may be convinced to invest in advanced microscopy systems, they may be reluctant to further invest in database servers, computers, and image analysis software. An online image storage and processing pipeline strategy on a secure server facilitates data manipulation without overloading local resources, as would be the case with data transfer over local networks, for example. A practical and simple alternative solution that should not be overlooked consists of transferring data using physical drives. In addition to the equipment, most biologists lack the competencies to develop a functional data-handling pipeline for LSFM images without assistance from experts in computer science (Gibbs et al., 2021; Reynaud et al., 2015). Once again, collaboration with other teams or core facilities is often the best solution to access the required computing environment.

Computational treatment of LSFM images can be decomposed into two main steps. First, image preprocessing is required to compensate for imaging artifacts before stitching the tiles to obtain a volumetric dataset (Gibbs et al., 2021). The second step consists of extracting useful information from the image and often involves segmentation followed by quantitative measurement of segmented objects (Delgado-Rodriguez et al., 2022; Gibbs et al., 2021; Richardson et al., 2021). However, segmentation accuracy depends on image resolution and, even in optimal conditions, it may fail if the objects of interest are too close to each other. For instance, while nuclei segmentation may be possible in most biological tissues, it is much more challenging within organoids in which the cells are much more compacted (Andilla et al., 2017). Similarly, while sparse labeling allows tracing long-range neuronal projections in the central and peripheral nervous system, this may not be possible in densely labeled areas (Ariel, 2018; Newmaster et al., 2022; Richardson et al., 2021). Importantly, image analysis requirements should be identified during the earliest steps of the project since they should specify the requirements for sample preparation and acquisition parameters. No existing algorithm can compensate for an insufficiently cleared or poorly labeled sample or a suboptimal acquisition (Newmaster et al., 2022).

Computational tools have multiplied in parallel with the development of tissue-clearing techniques and LSFM systems. Both open-source and commercial software tools are available, the latter being extremely expensive but usually more user-friendly. In both categories, machine learning is becoming unavoidable to perform complex operations with high precision. We will not detail here the existing solutions, but we refer the readers wishing to determine which software to choose for their specific application to the excellent review published by Gibbs et al. (2021). Among the latest innovations, the SyGlass software developed by the company IstoVisio has made possible a real interactive immersion in 3D LSFM images, thanks to virtual reality adapted to head-mounted display technology, thus facilitating visualization and annotation (Pidhorskyi et al., 2018 *Preprint*).

## Conclusion

Throughout this review, we have emphasized all the challenges faced by research teams aiming to develop a 3D imaging pipeline with LSFM from sample preparation to image analysis. Of course, our goal is not to discourage potential LSFM users but rather to help them avoid some identified pitfalls and find their way through the profusion of articles and protocols on the matter. The online survey we conducted in our network identified a significant proportion of users who were not satisfied with their LSFM experience. In addition to technical issues that are often mentioned as the main obstacle, such as the system’s limits, sample labeling and clearing, or image analysis, we believe that the main difference between successful and unsuccessful projects resides in the biological question. Movies starring rotating 3D reconstructions of entire organs are extremely appealing. However, the biological insight that can be gained from these movies is very limited if the initial question is not precisely defined and if its compatibility with LSFM imaging is not assessed before starting the experiments. LSFM is a powerful tool for morphologically characterizing anatomical structures, quantifying objects as small as individual cells provided they are sufficiently spaced apart from each other, and tracing filamentous structures such as blood vessels, airways, or neuronal projections (Ariel, 2018; Newmaster et al., 2022; Richardson et al., 2021). Due to the limited magnification and resolution compared with other systems, it is usually not adequate for subcellular imaging or for quantifying or tracing small structures in densely labeled areas (Ariel, 2018). Increasing attention has been given by the scientific community to the quality control checks required in light microscopy to ensure the accuracy, reproducibility, and comparability of image acquisitions, including in LSFM (see text box). However, since multiple factors associated with sample preparation and positioning influence the collected signal (independently of the acquisition parameters), comparing fluorescent intensities between different samples may not provide biologically relevant information (Hobson et al., 2022). Biologists tend to consider image acquisition as a prerequisite to determine which kind of image analysis can be performed. However, it is crucial for sample preparation and acquisition parameters to be tailored to image analysis requirements, and not the opposite. Keeping the entire pipeline in mind from the beginning of a project is an interdisciplinary challenge, as it involves competencies in biology, chemistry, physics, and computer science. Fortunately, a very dynamic and open community of scientists from various fields interested in tissue clearing and LSFM imaging has developed and can assist new LSFM users. In addition to workshops and conferences which are regularly organized, online repositories allow researchers to share their protocols and experience (Table 1). Like others, we believe that these interactions with the community are key to an LSFM experience reaching its full potential. New technologies are being developed to push forward the limits of the actual systems in terms of resolution (Daetwyler and Fiolka, 2023; Delgado-Rodriguez et al., 2022; Olarte et al., 2018). At the time of writing, these techniques are not yet accessible to most users, but they will likely reshape the landscape of LSFM imaging in the near future.

Quality control (QC) checks in LSFM

 The QC of light microscopes and the reproducibility of data collected by these machines are two subjects to which the utmost attention should be paid. Considering the various factors that affect data acquisition at different stages of the experimental workflow is crucial for obtaining accurate, reproducible, and interpretable data in imaging experiments (Boehm et al., 2021). The emulation generated by these topics in recent years demonstrates the interest given by the microscopist community, as shown by the creation of the international QUAREP-LiMi consortium (Quality Assessment and Reproducibility for Instruments and Images in Light Microscopy, https://quarep.org/; Nelson et al., 2021), and the work of the QC working group of the Réseau Technologique de Microscopie photonique de Fluorescence Multidimensionnelle, Centre National de la Recherche Scientifique, France (https://rtmfm.cnrs.fr/), published in a journal aimed at a wide biologist audience (Faklaris et al., 2022). LSFM should not escape the need to carry out these QC checks, although there is currently little work on this subject.

The vast majority of LSFM images are acquired on commercial systems and in multiuser environments within core facilities. In this context, it is crucial to regularly carry out measurements to follow the health status of the microscope. However, the most exciting LSFM applications nowadays still rely on custom solutions (Power and Huisken, 2017). The scientists who develop these systems can be expected to be rigorous in the settings that involve QC, although precise protocols dedicated to LSFM for carrying out the various QC measurements have yet to be defined. However, many of those can already be made based on previously published works, such as:

**The power and stability of illumination sources.** These two factors are essential, particularly when comparing fluorescence intensities between images, in time-lapse experiments, or when conducting experiments at different independent times or using different microscopes (Gaudreault et al., 2022).
**The field homogeneity.** For accurate quantitative imaging, it is crucial to ensure uniform illumination across the detection FOV, especially in LSFM for a large set of images encompassing the entire sample. When the illumination is not consistent over a wide area, the fluorescence intensities no longer accurately represent the intrinsic fluorescence, but instead, they become influenced by the specific location within the image (Brown et al., 2015; Hobson et al., 2022).
**The coregistration in x, y, and z.** This parameter is essential for image registration. If the alignment of the system varies in terms of both lateral or axial dimensions across the detectors or filter cubes/filter wheel positions used in an imaging experiment, the overlay of these images will not exhibit ideal coregistration even for perfectly coregistered samples. The chromatic corrections of the imaging lens are crucial here too (Faklaris et al., 2022; Preibisch et al., 2010; Stack et al., 2011).
**The stage positioning repeatability and xyz drift.** In the context of multidimensional acquisitions that involve multiple planes, positions, and time points, particularly in LSFM, stage stability holds significant importance (Faklaris et al., 2022).
**The camera noises.** The final element in the acquisition chain is the detector, of which cameras account for the vast majority in LSFM. The noise generated by these detectors needs to be monitored regularly to avoid drift, which could affect the reproducibility of acquisitions (Faklaris et al., 2022).

However, other measurement protocols of great interest and very specific to LSFM are still missing since no simple low-cost protocols are available, such as:

**The point spread function,** characterizing the resolution of the imaging system (Duocastella et al., 2017; Hobson et al., 2022; Power and Huisken, 2017; Vladimirov et al., 2023 *Preprint*).
**The homogeneity of the light sheet thickness,** allowing regular depth of field excitation across the whole detection FOV (Feng et al., 2021; Gao, 2015a, 2015b; Hedde and Gratton, 2018; Hobson et al., 2022).
**The alignment of the light sheet to the focal plane of the detection and coregistration of excitation sheets in double-sided illumination systems.** Any misalignment between the excitation sheets would lead to a loss of the precision of the optical sectioning intrinsic to the LSFM. Any misalignment between the excitation sheet(s) and the detection focal plane would have an impact on the intensity and location of the emitted fluorescence, thus altering any quantification (Feng et al., 2021; Hobson et al., 2022; Icha et al., 2016; Power and Huisken, 2017; Schmied and Tomancak, 2016).
**The match between the excitation and detection lens(es)** by ensuring that the two optical pathways are orthogonally coaligned and by adapting the numerical apertures of the excitation and detection lenses (Huisken and Stainier, 2007; Jahr et al., 2016; Olarte et al., 2018).
**The appropriate corrections of the lenses** (excitation and detection if not the same) that are chosen according to the imaging media and their respective refractive indexes (Hobson et al., 2022; Power and Huisken, 2017).

To go a step further than the QC aspect of LSFM, it is necessary to consider the characteristics of this technique for quantitative experiments (Hobson et al., 2022). From understanding how the system works—which is where QC comes in extremely useful—to archiving the raw and processed data, the rigorous design of the total experimental workflow will play a crucial role in the success of the experiments carried out through LSFM (Brown, 2007; Jost and Waters, 2019; Wait et al., 2020).
